# Has “Erasing” Made Things Clearer? Commentary on Schmidt, Liefooghe & De Houwer (2020, JoC): “An Episodic Model of Task Switching Effects: Erasing the Homunculus from Memory”

**DOI:** 10.5334/joc.111

**Published:** 2020-09-10

**Authors:** Iring Koch, Aureliu Lavric

**Affiliations:** 1 Institute of Psychology, RWTH Aachen University, Aachen, DE; 2 Department of Psychology, University of Exeter, Exeter, UK

**Keywords:** Attention, Cognitive Control, Mathematical modelling

## Abstract

Commentary on Schmidt, Liefooghe & De Houwer (2020, JoC): “An episodic model of task switching effects: Erasing the homunculus from memory”.

Schmidt, Liefooghe, and De Houwer (2020 [this volume]) present the Parallel Episodic Processing (PEP) model, originally developed to simulate practice, contingency, congruency, conflict adaptation and other effects in variants of the Stroop paradigm (Schmidt, 2013; Schmidt, De Houwer, & Rothermund, 2016), and described by the authors as “an exemplar-based memory model”. The new version was adapted to simulate performance in the most common variant of the task-switching paradigm – task cueing – with an emphasis on immediate repetitions of task cues, stimuli, and responses (henceforth: ‘C/S/R-rep’), which are well known to potentially inflate performance measures of the task ‘switch cost’, despite being empirically separable from the switch cost. In this commentary, we first summarise the key outcomes of the modelling and then turn to the key aspects of the author’s conclusions.

To attempt a summary of what the model does/doesn’t do, we asked some basic questions – first about the modelling outcomes. Does the model produce C/S/R-rep effects? Yes – and it seems more apt at doing so than several other task-switching models. Can the model account for the response congruence effect found in task-switching experiments? Yes – and it can also account for a greater effect of congruence during switches. Since the authors’ aim to demonstrate that “feature integration biases are the primary means via which the PEP model explains task switching behaviour, rather than task-set control”, we also ask: does “exemplar-based memory” explain the entire task switch cost? Here the answer is ‘No’ – part of the model’s switch cost is irreducible to C/S/R-rep effects. Likewise, does the model identify a novel (not hitherto suggested) mechanism for the portion of the switch cost irreducible to C/S/R-rep effects? No – this portion of the switch cost is produced in the model by persisting (inertial) activation of task “Goal” nodes. Finally, given the authors’ view (see below) that their model is incompatible with ‘task-set reconfiguration’ (TSR), we also ask if they attempted to model the reduction in switch cost with increasing the cue-stimulus interval – widely seen as the best evidence of TSR? No.

Thus, the model simulates the effects of C/S/R-rep on performance in task-switching experiments (known for ~15 years, but not hitherto comprehensively modelled in this domain), and produces response congruence effects and the congruence by switch interaction. Yet, a non-trivial portion of the switch cost irreducible to C/S/R-rep effects is modelled essentially as ‘task-set inertia’ (Allport, Styles, & Hsieh, 1994), and widely-accepted evidence of TSR (i.e., the reduction in switch cost with preparation) is not accounted for.

Turning to the authors’ own interpretation, the paper’s narrative revolves around the authors’ belief that (both in their modelling and in empirical data) “…the switch cost is mainly induced by feature integration biases and much less by task-set control” – which in turn relies on the premise that the ‘accepted norm’ in task-cueing experiments is to repeat the cue when the task is repeated. But, cue repetitions can be easily avoided (by using more than one cue per task and changing the cue even when the task repeats) – and we note that, both in the PEP model and in the empirical data it simulates, omitting cue repetitions decisively changes the balance of switch costs attributable to “feature integration” (S/R-rep effects) vs. “task-set control” in favour of the latter. Given the large (and growing) number of task-cueing studies where the switch cost (and its reduction) has been measured without repeating cues, we wonder if the far-reaching conclusion that “These feature integration biases are the primary means via which the PEP model explains task switching behaviour, rather than task-set control” might be premature. After all, the model’s “episodic memory” can only learn about repetitions (e.g., of cues) if there are such repetitions in the first place.

To us the narrative also suggests that the authors are on a sort of ‘mission’ against cognitive control of task-set, though, curiously, “…without denying the important role of cognitive control in executing a task more generally…”, and whilst admitting seemingly reluctantly that “The Goal nodes, along with their connectivity to other (e.g., cue Input and Decision) nodes, might be argued to be a computational implementation of task-sets”. The authors seem especially eager to “erase” (our reference to their title) their notion of TSR, which they define as “The idea that the task-set must be ‘reprogrammed’ as a discrete stage before preceding to stimulus processing” (Appendix A: List of Terminology). That this definition seems to represent more of a strawman than the current TSR framework is obvious here: “It is certainly not obvious that some part of TSR cannot be carried out in parallel with task-specific processes” (Monsell, 2017; p. 37). Indeed, it has been known since Logan and Gordon’s (2001) ECTVA model that computations compatible with TSR (‘parameter transmission’ in ECTVA) can be ‘dynamic’ and formally tractable.

Indeed, we wonder whether the authors may have overlooked TSR-compatible components or parameters in their own model. Consider how the model would produce the (amply evidenced) reduction in switch cost (RISC) with increasing the cue-stimulus interval – in conditions where task cues are not repeated and the response-stimulus interval (and hence “Goal” inertia) is controlled (i.e., constant). One component of the model which may produce a RISC effect is “lateral” “Goal”-“Goal” inhibition: during a longer cue-stimulus interval the relevant “Goal” node might have more time to inhibit the irrelevant “Goal” node, thus overcoming (in part) “Goal” persistence (task-set inertia). Indeed, there is substantial evidence to suggest that task-set inhibition may play a central role in task-set control (e.g., Koch, Gade, Schuch, & Philipp, 2010; see Koch, Poljac, Müller & Kiesel, 2018, for a more recent review). Another parameter to consider is the “Goal”-“Decision” “sensitization” parameter – it is currently fixed, but could be given a temporal dynamic; in conjunction with the non-linear activation function of “Decision” nodes this may conceivably produce the RISC effect.

We hope these considerations may give the authors some second thoughts regarding their conclusion that “…it seems clear that the PEP model is not consistent with the task-set reconfiguration account”. The authors may object that this comes down to terminological ambiguity, e.g., “As with many other concepts we discuss in this paper, this idea might depend on how one defines cognitive control”. Whilst we share the authors’ frustration with all-encompassing ‘umbrella’ terms such as ‘task-set control’, we are doubtful that denying hypothetical representations and processes denoted by them is the solution. As already discussed, it is obvious that C/S/R-rep effects do not explain away the switch cost. Furthermore, ERP research mentioned by the authors (Lavric, Mizon, & Monsell, 2008), and numerous other ERP studies, found large and highly reproducible switch-repeat differences which cannot, in principle, be due to C/S/R-rep, because those switch-repeat ERP effects occurred before the stimulus – at ~0.5s following the cue that (in at least several studies) was never repeated. If the authors become interested in the RISC effect – there is the formidable challenge of explaining why preparation substantially reduces, but does not eliminate, the switch cost, leaving the ‘residual switch cost’. And, of course, there are the other effects in task-switching, too numerous to discuss here (Kiesel, Steinhauser, Wendt, Falkenstein, Jost, Philipp, & Koch, 2010) – plenty of scope for further modelling.

## References

[1] Allport, A., Styles, E. A., & Hsieh, S. (1994). Shifting intentional set: Exploring the dynamic control of tasks In C. Umiltà & M. Moscovitch (Eds.), Attention and Performance XV: Conscious and nonconscious information processing (pp. 421–452). Cambridge, MA: MIT Press.

[2] Kiesel, A., Steinhauser, M., Wendt, M., Falkenstein, M., Jost, K., Philipp, A. M., & Koch, I. (2010). Control and interference in task switching—A review. Psychological Bulletin, 136, 849–874. DOI: 10.1037/a0019842 20804238

[3] Koch, I., Gade, M., Schuch, S., & Philipp, A. M. (2010). The role of inhibition in task switching: A review. Psychonomic Bulletin & Review, 17, 1–14. DOI: 10.3758/PBR.17.1.1 20081154

[4] Koch, I., Poljac, E., Müller, H., & Kiesel, A. (2018). Cognitive structure, flexibility, and plasticity in human multitasking – An integrative review of dual-task and task-switching research. Psychological Bulletin, 144, 557–583. DOI: 10.1037/bul0000144 29517261

[5] Lavric, A., Mizon, G. A., & Monsell, S. (2008). Neurophysiological signature of effective anticipatory task-set control: a task-switching investigation. European Journal of Neuroscience, 28, 1016–1029. DOI: 10.1111/j.1460-9568.2008.06372.x 18717737

[6] Logan, G. D., & Gordon, R. D. (2001). Executive control of visual attention in dual-task situations. Psychological Review, 108, 393–434. DOI: 10.1037/0033-295X.108.2.393 11381835

[7] Monsell, S. (2017). Task set regulation In T. Egner (Ed.), The Wiley handbook of cognitive control (pp. 29–49). Wiley-Blackwell DOI: 10.1002/9781118920497.ch2

[8] Schmidt, J. R. (2013). The Parallel Episodic Processing (PEP) model: Dissociating contingency and conflict adaptation in the item-specific proportion congruent paradigm. Acta Psychologica, 142, 119–126. DOI: 10.1016/j.actpsy.2012.11.004 23261421

[9] Schmidt, J. R., De Houwer, J., & Rothermund, K. (2016). The Parallel Episodic Processing (PEP) Model 2.0: A single computational model of stimulus-response binding, contingency learning, power curves, and mixing costs. Cognitive Psychology, 91, 82–108. DOI: 10.1016/j.cogpsych.2016.10.004 27821256

[10] Schmidt, J. R., Liefooghe, B., & De Houwer, J. (2020). An episodic model of task switching effects: Erasing the homunculus from memory. Journal of Cognition. 3(1): 22, pp. 1–38. DOI: 10.5334/joc.97 PMC7485406 32964181

